# *Caenorhabditis elegans* as an In Vivo Infection–Rescue Model for Early-Stage Antimicrobial Screening Against Gram-Positive and Gram-Negative Bacteria

**DOI:** 10.3390/antibiotics15100946

**Published:** 2026-09-23

**Authors:** Salman Mirza, Jo-Ann McClure, John M. Conly, Kunyan Zhang

**Affiliations:** 1 Department of Pathology & Laboratory Medicine, University of Calgary and Alberta Health Services, Calgary, AB T2N 4N1, Canada; salman.mirza1@ucalgary.ca (S.M.); jammcclu@ucalgary.ca (J.-A.M.); john.conly@albertahealthservices.ca (J.M.C.); 2 Department of Microbiology, Immunology and Infectious Diseases, University of Calgary, Calgary, AB T2N 4N1, Canada; 3 Centre for Antimicrobial Resistance, Alberta Health Services/Alberta Precision Laboratories/University of Calgary, Calgary, AB T2N 4N1, Canada; 4 Department of Medicine, University of Calgary and Alberta Health Services, Calgary, AB T2N 4N1, Canada; 5 The Calvin, Phoebe and Joan Snyder Institute for Chronic Diseases, University of Calgary and Alberta Health Services, Calgary, AB T2N 4N1, Canada

**Keywords:** *Caenorhabditis elegans*, in vivo infection, infection–rescue assay, antibiotic discovery, antimicrobial resistance, antimicrobial screening, whole-animal model, Gram-positive bacteria, Gram-negative bacteria

## Abstract

**Background/Objectives**: The growing global burden of antimicrobial resistance highlights the need for whole-animal screening platforms that better reflect in vivo determinants of antimicrobial efficacy. A *Caenorhabditis elegans* infection–rescue model previously developed for antibiotic screening against *Enterococcus faecalis* was adapted to evaluate additional clinically relevant Gram-positive and Gram-negative bacteria. **Methods**: L4-stage AU37 worms were infected with *Staphylococcus aureus* 2406, *E. faecalis* ATCC51299, *Escherichia coli* CFT073, and *Pseudomonas aeruginosa* PAO1. Pathogen-specific infection durations were optimized using conventional antibiotic treatments, including vancomycin for *S. aureus* and gentamicin for *E. faecalis*, *E. coli*, and *P. aeruginosa*. Following antibiotic treatment, pathogen-specific mean rescue rates (MRR) were determined. Assay performance was then validated using positive- and negative-control antibiotics selected based on contrasting susceptibility profiles. **Results**: Optimized antibiotic-mediated rescue occurred at pathogen-specific time points following treatment with vancomycin for *S. aureus* and gentamicin for the remaining pathogens, 48 h for *S. aureus* (MRR, 92.6% ± 3.5%), 72 h for *E. faecalis* (89.4% ± 3.6%), 4 h for *E. coli* (94.5% ± 3.6%), and 72 h for *P. aeruginosa* (85.5% ± 9.1%) following treatment with conventional antibiotics, with all treated groups differing significantly from controls (*p* < 0.001). Positive-control antibiotics, including chloramphenicol, tetracycline, and amikacin, produced strong rescue at the optimized infection time points. In contrast, negative-control antibiotics, including erythromycin and chloramphenicol, produced minimal rescue. **Conclusions**: These data show that the *C. elegans* infection–rescue model can distinguish effective from ineffective antibiotic treatment across the four tested bacterial infection conditions and may serve as a useful early-stage platform for antimicrobial screening.

## 1. Introduction

Antimicrobial resistance (AMR) represents one of the most significant threats to global public health, undermining decades of progress in the treatment of bacterial infections [1,2]. Although the introduction of antimicrobials drastically reduced infection-associated mortality, the widespread and often inappropriate use of these antibiotics has driven the rapid emergence and dissemination of resistance across bacterial pathogens [3,4]. Resistance determinants are frequently acquired through horizontal gene transfer and mobilized via plasmids, transposons, and other mobile genetic elements, enabling their spread within and between species [5,6,7,8]. As a result, infections caused by multidrug-resistant (MDR) organisms are increasingly difficult to treat, highlighting the urgent need for novel antibiotics and improved discovery strategies.

Several clinically important bacterial pathogens exemplify the challenges posed by AMR. *Staphylococcus aureus*, a Gram-positive pathogen, is responsible for a broad spectrum of diseases ranging from superficial skin infections to life-threatening systemic infections such as bacteremia, pneumonia, and endocarditis [9,10,11]. The emergence of methicillin-resistant *S. aureus* (MRSA), driven primarily by acquisition of the *mecA* gene, which encodes the low-affinity penicillin-binding protein PBP2a, has rendered most β-lactam antibiotics ineffective [9,12,13,14]. Similarly, *Enterococcus faecalis* is a major cause of healthcare-associated infections, including urinary tract infections and endocarditis, and frequently acquires resistance to multiple antimicrobial compounds [15,16,17]. Of particular concern is vancomycin-resistant enterococci (VRE), which arise through acquisition of the *vanA* gene cluster located on transferable elements such as Tn1546, facilitating dissemination of resistance [15,18,19,20].

Gram-negative pathogens further complicate the AMR concern. *Escherichia coli*, one of the most common pathogens, has evolved diverse resistance mechanisms, including β-lactamase production, efflux pump activation, altered membrane permeability, and target modification [21,22,23]. *Pseudomonas aeruginosa*, a leading cause of opportunistic infections in hospitalized and immunocompromised patients, exhibits remarkable genomic plasticity and intrinsic resistance, allowing rapid adaptation under antibiotic pressure and frequent emergence of MDR phenotypes [24,25,26]. Together, this increase in resistance underscores the need for antimicrobial screening models that are both rapid and high-throughput, capable of capturing biologically relevant determinants of efficacy across diverse pathogens.

Traditionally, antimicrobial discovery has relied heavily on in vitro assays that measured outcomes, such as growth inhibition under controlled laboratory conditions. While these assays are valuable for initial screening, they overlook other factors, such as host-associated factors, compound toxicity, bioavailability, and pathogen behavior in vivo. Consequently, compounds that appear promising in vitro may subsequently fail in vivo, and potential compounds with favorable in vivo properties may also be overlooked in vitro.

The nematode *Caenorhabditis elegans* has emerged as a useful model organism for studying innate immunity, host–pathogen interactions, and antimicrobial activity because it is inexpensive to maintain, rapidly propagated, genetically tractable, transparent, and suitable for scalable survival-based infection assays [27,28,29]. As a free-living and bacterial feeding animal, *C. elegans* can be infected by several clinically relevant human pathogens, including *S. aureus*, allowing bacterial virulence mechanisms and antibiotic-mediated rescue to be studied within a live host environment. Importantly, some pathogen-specific virulence determinants identified in *C. elegans* show parallels with those observed in mammalian infection models [28,30,31,32].

The *C. elegans* infection–rescue assay originally developed by Moy et al. using *E. faecalis* established a powerful whole-animal model for identifying antimicrobial compounds with in vivo efficacy, allowing detection of both bactericidal agents and anti-infective mechanisms, including those that were not previously captured in vitro [33,34,35]. Moy et al. specifically selected *E. faecalis* because it forms a persistent lethal intestinal infection in *C. elegans*, enabling quantitative assessment of antimicrobial-mediated rescue within a live host system [33].

Although previous studies have shown that significant human pathogens such as *S. aureus*, *P. aeruginosa*, and *E. coli* can infect and kill *C. elegans*, these studies largely focused on host–pathogen interactions rather than systematic antimicrobial rescue across diverse bacterial species [24,25,26,36].

Therefore, whether the *C. elegans* infection–rescue framework can be extended beyond *E. faecalis* to evaluate antibiotic-mediated rescue across biologically distinct Gram-positive and Gram-negative pathogens remains unclear. Given that Gram-positive and Gram-negative bacteria exhibit distinct cell wall architectures, virulence strategies, and intrinsic antibiotic resistance mechanisms [37,38,39], evaluating antimicrobial efficacy across these pathogens may help address an important gap in the implementation of such whole-animal screening approaches.

In our study, we adapted the *C. elegans* infection–rescue framework to include clinically relevant Gram-positive (*S. aureus*, *E. faecalis*) and Gram-negative (*E. coli*, *P. aeruginosa*) pathogens using the temperature-sensitive, immune-deficient AU37 (*glp-4*; *sek-1*) strain previously implemented by Moy et al. [33,34,35]. The *glp-4* mutation confers temperature-sensitive sterility, reducing progeny-associated interference during survival scoring, while the *sek-1* mutation impairs p38 MAPK-mediated innate immune signaling, increasing susceptibility to bacterial infection and improving assay sensitivity [33,34,40,41]. We performed assay-specific antibiotic growth inhibition testing, optimized pathogen-specific infection durations, selected positive- and negative-control antibiotics based on contrasting susceptibility profiles, and quantified antibiotic-mediated rescue using mean rescue rate (MRR). Together, this approach allowed us to determine whether the infection–rescue assay could distinguish effective from ineffective antibiotic treatment across the tested Gram-positive and Gram-negative bacterial pathogens.

## 2. Results

### 2.1. Assay-Specific Growth Inhibition Testing of Conventional Antibiotics

To establish antibiotic concentrations for the *C. elegans* infection assay, broth microdilution-based in vitro growth inhibition testing was performed under the assay conditions used in this study (Table 1). These experiments were used to identify working antibiotic concentrations capable of suppressing bacterial growth during subsequent worm infection–rescue assays and were not intended to define clinical susceptibility breakpoints. Under these conditions, vancomycin inhibited growth of *S. aureus* 2406 at 5 µg/mL. Gentamicin inhibited growth of *E. faecalis* ATCC 51299 and *E. coli* CFT073 at 25 µg/mL, while *P. aeruginosa* PAO1 exhibited inhibition at 6.25 µg/mL. For each pathogen, the working antibiotic concentration used in the infection–rescue assay was then selected as two-fold above the assay-specific growth-inhibitory concentration. The corresponding concentrations selected for the infection–rescue assay were 10 µg/mL vancomycin for *S. aureus* and 50 µg/mL gentamicin for *E. faecalis* and *E. coli*, 12.5 µg/mL gentamicin for *P. aeruginosa*.

### 2.2. Pathogen-Specific Infection Durations Determine Optimized Antibiotic-Mediated Rescue Windows

To determine the optimal infection duration for each pathogen, untreated mortality and antibiotic-mediated rescue were assessed across multiple infection time points (Figure 1A–D). The optimized infection duration was determined as the time point that maximized pathogen exposure while maintaining a high mean rescue rate (MRR) and allowing reliable survival scoring. Using these criteria, the selected infection durations were 48 h for *S. aureus* 2406, 72 h for *E. faecalis* ATCC 51299, 4 h for *E. coli* CFT073, and 72 h for *P. aeruginosa* PAO1. These pathogen-specific time points were used for subsequent assay-validation experiments.

Infection with Gram-positive *S. aureus* 2406 resulted in rapid nematode mortality, with complete death observed by 48 h post-infection in untreated controls, whereas antibiotic treatment at this time point produced optimal rescue (MRR, 92.6% ± 3.5%) (Figure 1A). In contrast, *E. faecalis* ATCC51299 exhibited a slower, progressive killing trajectory, with optimal rescue observed at 72 h post-infection (MRR, 89.4% ± 3.6%) (Figure 1B).

The Gram-negative pathogen *E. coli* CFT073 induced rapid nematocidal activity, resulting in complete mortality within 2–6 h post-infection. Among the tested time points, optimized rescue was observed at 4 h (MRR, 94.5% ± 3.6%) (Figure 1C). *P. aeruginosa* PAO1 displayed a more prolonged infection course, with near-complete mortality by 72 h and optimized rescue was observed at this time point (MRR, 85.5% ± 9.1%) (Figure 1D).

### 2.3. Selection of Positive- and Negative-Control Antibiotics for Assay Validation

To validate whether the *C. elegans* infection–rescue assay could distinguish effective from ineffective antibiotic exposure, one positive-control and one negative-control antibiotic were selected for each bacterial strain based on contrasting susceptibility profiles determined by Kirby–Bauer disk diffusion testing (see Table 2 and Table S1). These antibiotic–pathogen pairs were selected solely for assay validation and were not intended to represent clinically recommended treatment combinations. The selected positive-control antibiotics were chloramphenicol for *S. aureus* 2406, tetracycline for *E. faecalis* ATCC 51299 and *E. coli* CFT073, and amikacin for *P. aeruginosa* PAO1. The selected negative-control antibiotics were erythromycin for *S. aureus* 2406 and *E. coli* CFT073, and chloramphenicol for *E. faecalis* ATCC 51299 and *P. aeruginosa* PAO1.

### 2.4. Validation of the C. elegans Infection–Rescue Assay Using Positive- and Negative-Control Antibiotics

Treatment of infected worms with positive- and negative-control antibiotics produced clear, pathogen-specific differences in rescue outcomes at the optimized infection durations (Figure 2A–D).

In *S. aureus* 2406 infections, treatment with the positive-control (susceptible) antibiotic chloramphenicol (6.25 µg/mL) at 48 h post-infection resulted in strong rescue (MRR, 92.0% ± 6.3%). In contrast, treatment with the negative-control (resistant) erythromycin produced minimal rescue (MRR, 2.8%), similar to untreated infection controls (Figure 2A).

In *E. faecalis* ATCC51299 infections, treatment with the positive-control antibiotic tetracycline at 72 h post-infection produced strong rescue (MRR, 90.8% ± 4.6%). In contrast, treatment with the negative-control antibiotic chloramphenicol resulted in poor rescue (MRR, 7.5%) (Figure 2B).

In *E. coli* CFT073 infections, treatment with the positive-control antibiotic tetracycline at 4 h post-infection produced near-complete rescue (MRR, 95.9% ± 2.6%). In contrast, treatment with the negative-control antibiotic erythromycin failed to rescue infected worms, resulting in mortality similar to untreated controls (MRR, 1.3%) (Figure 2C).

In *P. aeruginosa* PAO1 infections, treatment with the positive-control antibiotic amikacin at 72 h post-infection resulted in strong rescue (MRR, 90.5% ± 3.6%). In contrast, treatment with the negative-control antibiotic chloramphenicol produced minimal rescue, with near-complete worm mortality observed (MRR, 5.0%) (Figure 2D).

## 3. Discussion

The growing burden of antimicrobial resistance (AMR) underscores the need for screening platforms that extend beyond traditional in vitro growth inhibition assays and better reflect biologically relevant determinants of antimicrobial efficacy [30,31,32,42]. Early studies established *C. elegans* as a whole-animal model for investigating bacterial virulence and host–pathogen interactions, demonstrating that clinically relevant pathogens such as *E. faecalis*, could colonize, infect and ultimately kill the nematode through virulence-associated mechanisms [43,44].

Building upon such foundational virulence studies, Moy et al. later developed a high-throughput infection–rescue framework using *E. faecalis*, specifically selecting this organism because of its ability to establish persistent intestinal colonization and reproducible killing kinetics within the worm model, thereby allowing reliable assessment of antibiotic-mediated rescue [33,34].

Subsequent studies further showed that clinically relevant pathogens such as *S. aureus*, *P. aeruginosa*, and *E. coli* produce distinct infection dynamics and host responses in *C. elegans*, supporting the utility of the nematode as a biologically reliable in vivo host system for studying pathogenesis [43,44,45,46].

The novelty of the present study lies in adapting a previously *E. faecalis*-focused *C. elegans* infection–rescue framework to multiple clinically relevant Gram-positive and Gram-negative bacterial pathogens. While earlier studies established *C. elegans* as a host model for bacterial virulence and host–pathogen interactions, fewer studies have systematically optimized pathogen-specific infection durations for antibiotic-mediated rescue across biologically distinct bacterial species. By defining rescue windows for *S. aureus*, *E. faecalis*, *E. coli*, and *P. aeruginosa*, this study expands the use of the infection–rescue assay as an early-stage antimicrobial screening platform.

Our study incorporates the use of the AU37 (*glp-4*; *sek-1*) mutant host strain similarly to Moy et al. [33,34], which combines temperature-sensitive sterility with impaired innate immune signaling [47]. In contrast to wild-type Bristol N2 worms, which exhibit prolonged mortality timelines extending up to 7–9 days (Wu et al.) [48], the AU37 strain produced more rapid and synchronized infection dynamics. This enhanced susceptibility improves temporal resolution and clearer distinction between untreated death and antibiotic-mediated rescue. The *glp-4* mutation prevents internal hatching of progeny that can otherwise confound mortality assessment, while the *sek-1* mutation disrupts the conserved p38 MAPK immune pathway, increasing susceptibility to bacterial infection and improving assay sensitivity [33,34,43]. Notably, AU37 is not intended to fully replicate human immune responses or mammalian pharmacokinetics. Rather, it provides a sensitized whole-animal infection host that can support reproducible detection of pathogen-mediated killing and antibiotic-mediated rescue. Therefore, this model is best interpreted as an intermediate screening platform between conventional in vitro assays and more complex mammalian infection models.

A central finding of this study was that the optimal infection duration was highly pathogen dependent. *S. aureus* induced substantial nematode mortality within 48 h postinfection, whereas *E. faecalis* and *P. aeruginosa* showed slower, progressive killing trajectories, with optimized rescue observed at 72 h. In contrast, *E. coli* CFT073 produced unusually rapid killing kinetics, with near-complete mortality occurring within 2–6 h postinfection and optimized rescue observed at 4 h. These differences likely reflect pathogen-specific variation in colonization, persistence, growth kinetics, and virulence activation within the worm host. Importantly, these findings show that antibiotic-mediated rescue cannot be interpreted independently of infection timing. If treatment is applied too early, untreated mortality may be insufficient to detect rescue; if treatment is applied too late, host damage may limit rescue even when the antibiotic has activity. Therefore, pathogen-specific optimization is essential for reliable interpretation of *C. elegans* infection–rescue assays.

Previous studies have demonstrated that *S. aureus* accumulates within the *C. elegans* intestinal tract and induces killing through conserved virulence regulators and secreted factors including *agr*, *sarA*, α-hemolysin, and extracellular proteases [31,32,48]. Similarly, persistent intestinal colonization and progressive host damage have been well described for *E. faecalis* infections in *C. elegans*, whereas *P. aeruginosa* pathogenesis may involve prolonged host interaction and/or secreted virulence mechanisms depending on infection conditions [33,43,49,50]. These pathogen-specific mechanisms likely contribute to the different mortality patterns and rescue windows observed in the current study.

The unusually rapid killing observed with *E. coli* should be interpreted cautiously. One possible contributor is that, in our liquid assay, nalidixic acid was omitted from the *E. coli* infection condition because of its inhibitory activity against the pathogenic strain (see Supplementary Table S4), potentially permitting residual OP50 worm food bacteria to persist and proliferate during infection. These potential interactions between residual food-source bacteria and pathogenic *E. coli* may therefore have altered bacterial dynamics and accelerated mortality in untreated controls. This observation nonetheless remains speculative. Future studies measuring bacterial burden, intestinal colonization, and OP50 persistence will be needed to clarify the mechanism underlying the rapid mortality observed in this condition.

Previous work has also shown that nematode mortality requires live bacteria and is strongly influenced by bacterial strain and culture conditions, supporting the conclusion that active infection processes rather than simple bacterial presence drive pathogenicity [36]. More recent studies further show that *C. elegans* lacks canonical pattern-recognition receptors and instead detects infection through dysregulation in host physiology and stress responses [51,52]. Overall, this suggests that pathogen-specific growth kinetics and virulence mechanisms may distinctly initiate conserved innate defense pathways for the nematode.

Regardless of the underlying mechanisms, these pathogen-specific infection dynamics have direct implications for antimicrobial screening, as the timing of treatment determines the window in which differences between untreated mortality and antibiotic-mediated rescue can be detected. Without temporal optimization, effective antimicrobials may potentially be misclassified as inactive, and resistant compounds could appear effective due to suboptimal infection timing. Our findings show that assay performance is dependent on infection timing and that optimization must be tailored distinctly to each pathogen to ensure reliable interpretation of rescue outcomes.

Validation using positive- and negative-control antibiotics supported the ability of the assay to distinguish effective from ineffective antibiotic exposure. Across all four pathogens tested, positive-control antibiotics produced strong rescue, whereas negative-control antibiotics produced little or no rescue and resulted in mortality similar to untreated infected controls. These findings indicate that rescue in this model reflects antibiotic activity within the infected host environment rather than nonspecific toxicity or exposure effects. However, the present study did not directly measure bacterial burden, intestinal clearance, or antibiotic mode of action during rescue. Therefore, future work incorporating bacterial load measurements and colonization imaging will be required for determining whether rescue reflects reduced pathogen burden, altered virulence, improved host tolerance, or a combination of these effects. Additionally, several limitations should be acknowledged. Although *C. elegans* provides a useful bridge between conventional in vitro assays and more complex live-host infection models, it does not completely reproduce mammalian immunity, physiology, or pharmacokinetics. Therefore, antibiotic-mediated rescue in *C. elegans* should not be interpreted as direct evidence of clinical efficacy, and promising compounds identified using this platform would require validation in mammalian infection models. In addition, this study evaluated representative strains of each pathogen rather than broader strain panels, and bacterial burden, intestinal colonization, and antibiotic mode of action during rescue were not directly measured. The antibiotic concentrations used in this study were selected solely to support assay optimization and validation in the worm model and should not be interpreted as clinically relevant treatment concentrations. Future studies should expand this platform to include additional strain backgrounds, dose–response rescue curves, bacterial burden measurements, fluorescent colonization assays, antibiotic combination testing, and mammalian validation. To place the current model in context, we compared commonly used antimicrobial screening platforms with the *C. elegans* infection–rescue assay developed in this study (see Table 3).

In summary, this study supports the use of *C. elegans* infection–rescue assay as an early-stage platform for antimicrobial screening across the tested Gram-positive and Gram-negative bacterial pathogens. By integrating pathogen-specific infection timing with quantitative rescue outcomes, this model provides a rapid and scalable approach for distinguishing effective from ineffective antibiotic exposure in a live host setting. As antimicrobial resistance continues to challenge existing therapies, whole-animal screening approaches may contribute to the on-going efforts aimed at prioritizing candidate antibiotics, identifying novel therapeutics and strengthening antimicrobial development framework.

## 4. Materials and Methods

### 4.1. Bacterial Strains

The bacterial strains used in this study included *Staphylococcus aureus* 2406, *Enterococcus faecalis* ATCC 51299, *Escherichia coli* CFT073, and *Pseudomonas aeruginosa* PAO1. *S. aureus* 2406 is a Calgary clinical isolate originally recovered from a patient with necrotizing pneumonia during a local outbreak in 2004 as previously described by Wu et al. [53]. *E. faecalis* ATCC 51299 was obtained from the American Type Culture Collection (ATCC, Manassas, VA, USA) [54]. *E. coli* CFT073 was kindly provided by Dr. Rebekah DeVinney, and *P. aeruginosa* PAO1 was kindly provided by Dr. Shawn Lewenza, University of Calgary. All bacterial strains were cultured overnight at 37 °C in brain heart infusion (BHI) broth with aeration prior to experimental use.

### 4.2. Kirby–Bauer Disk Diffusion Susceptibility Testing

Antibiotic susceptibility profiles were determined using a disk diffusion method. Briefly, bacterial suspensions were prepared from overnight cultures, spread onto Mueller–Hinton agar plates, and antibiotic disks were applied to the agar surface. Plates were incubated at 37 °C for 18–24 h, after which inhibition zones were measured in millimeters. Susceptibility was interpreted as susceptible, intermediate, or resistant using the CLSI-based zone diameter interpretive criteria provided with the antibiotic disk reference chart. These results were used to select one positive-control and one negative-control antibiotic for each bacterial strain based on contrasting susceptibility profiles. The selected antibiotic–pathogen pairs were used solely to validate the *C. elegans* infection–rescue assay and were not intended to represent clinically recommended treatment combinations.

### 4.3. Assay-Specific Broth Microdilution Growth-Inhibition Testing

Broth microdilution-based growth inhibition testing was performed to identify working antibiotic concentrations for the *C. elegans* infection–rescue assay. Overnight bacterial cultures were diluted in brain heart infusion broth and adjusted to an absorbance at 600 nm of 0.08. CFU/mL was not directly quantified; therefore, these experiments were interpreted as absorbance-standardized, assay-specific growth-inhibition tests used to select working antibiotic concentrations for the *C. elegans* infection–rescue assay, rather than formal reference MIC assays. Antibiotics were prepared as two-fold serial dilutions in brain heart infusion broth. Vancomycin was tested against methicillin-resistant *S. aureus* 2406 over a concentration range of 1.25–20 µg/mL. Gentamicin was tested against *E. faecalis* ATCC 51299, *E. coli* CFT073, and *P. aeruginosa* PAO1 over a concentration range of 3.125–50 µg/mL. For assay-validation experiments, chloramphenicol, tetracycline, erythromycin, and amikacin were tested over a concentration range of 3.125–50 µg/mL, depending on the antibiotic–pathogen pair. Nalidixic acid was also tested over a concentration range of 5–40 µg/mL to determine whether it inhibited the bacterial strains used in the infection–rescue assay. Growth was assessed by visual inspection of turbidity relative to untreated growth controls and sterile media controls. The lowest antibiotic concentration showing no visible bacterial growth was recorded as the assay-specific growth-inhibitory concentration. These values were used to select working antibiotic concentrations (2-fold above) for subsequent *C. elegans* infection–rescue experiments and were not intended to define clinical susceptibility breakpoints.

### 4.4. C. elegans Strain

The temperature-sensitive, immunocompromised *C. elegans* strain AU37 (*glp-4*; *sek-1*) was used for all infection assays. Worms were maintained on nematode growth medium (NGM) agar plates seeded with *E. coli* OP50 as a food source according to standard protocols. Gravid adult hermaphrodites were synchronized by alkaline hypochlorite bleaching to obtain eggs, which were incubated at 15 °C and allowed to develop to the L4 larval stage prior to infection experiments. L4-stage worms were identified based on developmental timing following synchronization and confirmed by body size and vulval morphology using a stereomicroscope (Fisher Scientific, Waltham, MA, USA) [33,34]. *E. coli* OP50 was grown overnight at 37 °C, and 100 µL of the overnight culture was spread onto NGM agar plates. OP50-seeded NGM plates were incubated overnight before use for worm maintenance and synchronization. OP50 served as the standard food source for *C. elegans* and was not used as an experimental infection variable.

### 4.5. Nematode Infection Assay

L4-stage AU37 worms were transferred to tryptic soy agar (TSA) plates seeded with the bacterial pathogen of interest and incubated at 25 °C to initiate infection. Infection durations ranged from 2 to 72 h depending on the bacterial strain and experimental objective. Because pathogen lawns continued to grow during the infection period, bacterial exposure was standardized by using consistent culture preparation, agar medium, incubation temperature, and pathogen-specific infection duration across biological replicates rather than by a fixed CFU/mL at the time of worm transfer. For infection-duration optimization experiments, worms were treated with vancomycin for methicillin-resistant *S. aureus* 2406 and gentamicin for *E. faecalis* ATCC 51299, *E. coli* CFT073, and *P. aeruginosa* PAO1.

Following the designated infection period, worms were transferred to 24-well plates containing liquid assay medium composed of 80% M9 buffer and 20% BHI broth supplemented with the appropriate antibiotic at the selected working concentration. Working antibiotic concentrations were determined from assay-specific broth microdilution growth-inhibition testing. Control groups consisted of infected worms transferred to liquid assay medium without antibiotic treatment. For each experimental condition, approximately 70–80 worms were used per treatment group in each independent biological replicate.

For assay-validation experiments, one positive-control and one negative-control antibiotic were tested for each bacterial strain based on contrasting disk diffusion susceptibility profiles. The positive-control antibiotics were chloramphenicol for *S. aureus* 2406, tetracycline for *E. faecalis* ATCC 51299 and *E. coli* CFT073, and amikacin for *P. aeruginosa* PAO1. The negative-control antibiotics were erythromycin for *S. aureus* 2406 and *E. coli* CFT073 and chloramphenicol for *E. faecalis* ATCC 51299 and *P. aeruginosa* PAO1.

Nalidixic acid was included where appropriate to suppress residual *E. coli* OP50 growth during infection and rescue. Nalidixic acid was used at 10 µg/mL on TSA infection plates and in liquid rescue medium for *S. aureus*, *E. faecalis*, and *P. aeruginosa* experiments but was omitted from *E. coli* CFT073 experiments because preliminary growth-inhibition testing showed that nalidixic acid inhibited this strain (Supplementary Table S4).

Worm survival was scored after 24 h of antibiotic treatment using a stereomicroscope. Worms were classified as alive if they exhibited normal movement and sinusoidal morphology and as dead if they appeared straight, rigid and immobile. Sample images of live and dead worms are available in our previous publication [48]. A schematic overview of the experimental workflow is shown in Figure 3.

### 4.6. Data Analysis and Statistics

Worm mortality was calculated as the fraction of dead worms relative to the total number of worms in each treatment group. Mean rescue rate (MRR) was calculated for each independent biological replicate using the following formula:

MRR (%) = 100 − [(fraction death in antibiotic-treated group/fraction death in untreated infected control group) × 100].

The optimized infection duration for each pathogen was selected as the time point that maximized pathogen exposure while maintaining high untreated-control mortality and allowing feasible, reliable survival scoring. The biological replicate, rather than the individual worm, was treated as the statistical unit. Each biological replicate included approximately 70–80 worms per treatment group. Data are presented as mean ± standard deviation (SD) from four independent biological replicates.

Statistical analyses were performed using GraphPad Prism version 11.0.2 (GraphPad Software, Boston, MA, USA). For infection-duration optimization experiments, antibiotic-treated groups were compared with untreated infected controls at the same infection duration. For assay-validation experiments, MRR values were compared between positive- and negative-control antibiotics within each pathogen. Differences between two groups were analyzed using paired *t*-tests when treatment groups were matched within the same biological replicate experiment. A *p* value of <0.05 was considered statistically significant.

## Figures and Tables

**Figure 1:**
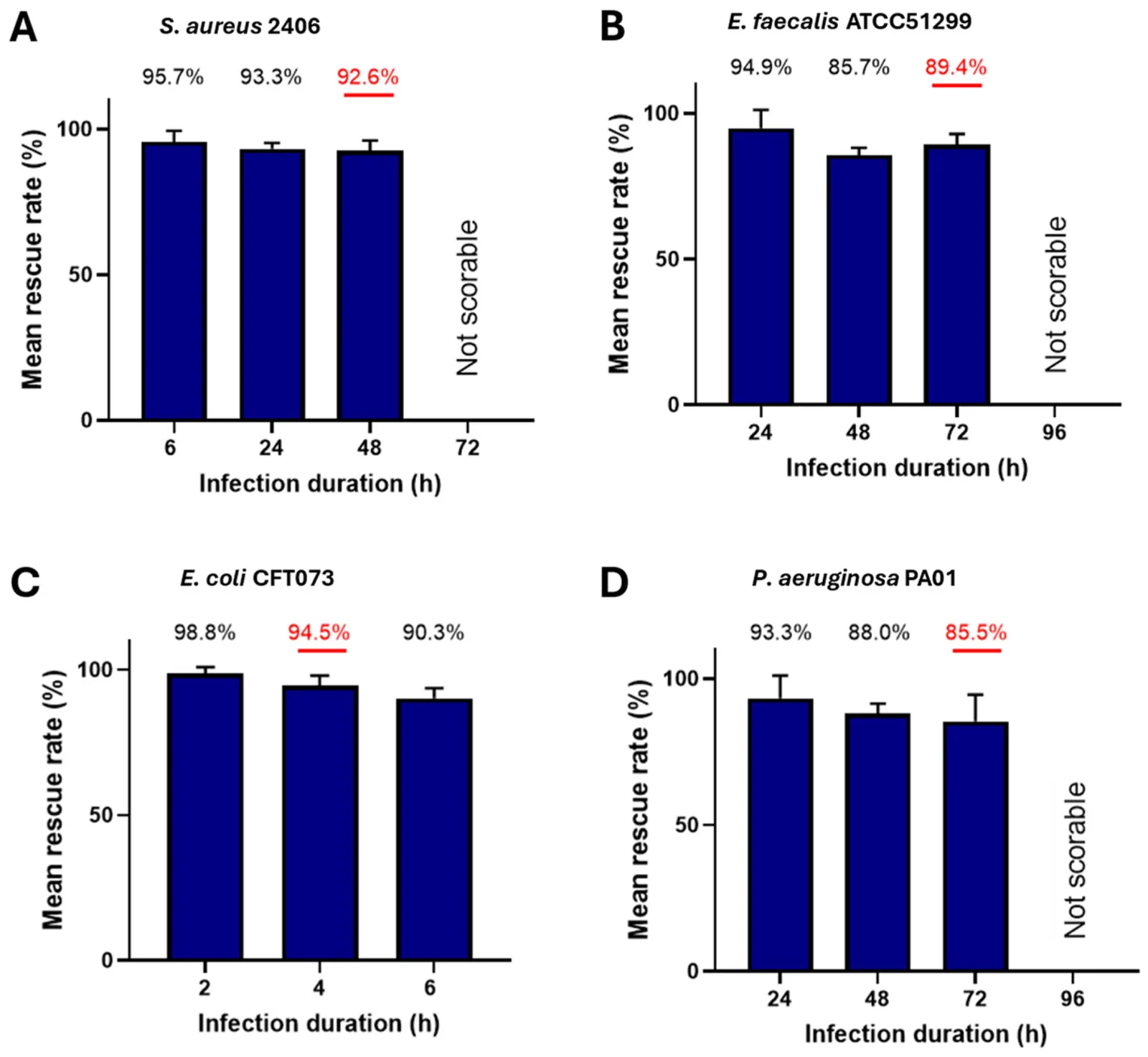
Pathogen-specific infection durations define optimized antibiotic-mediated rescue windows in the *C. elegans* infection–rescue assay. Mean rescue rate (MRR) of AU37 mutant *C. elegans* following infection with (**A**) *Staphylococcus aureus* 2406, (**B**) *Enterococcus faecalis* ATCC51299, (**C**) *Escherichia coli* CFT073, and (**D**) *Pseudomonas aeruginosa* PAO1 across multiple infection durations. Worms were infected for the indicated durations and treated with vancomycin for *S. aureus* or gentamicin for *E. faecalis*, *E. coli*, and *P. aeruginosa*. Bars represent mean MRR ± SD from four independent biological replicates. Values above bars indicate mean MRR for each infection duration. Red underlined values indicate the infection duration selected for subsequent assay-validation experiments based on the criteria described in the text. Time points at which survival could not be reliably assessed are indicated as not scorable.

**Figure 2:**
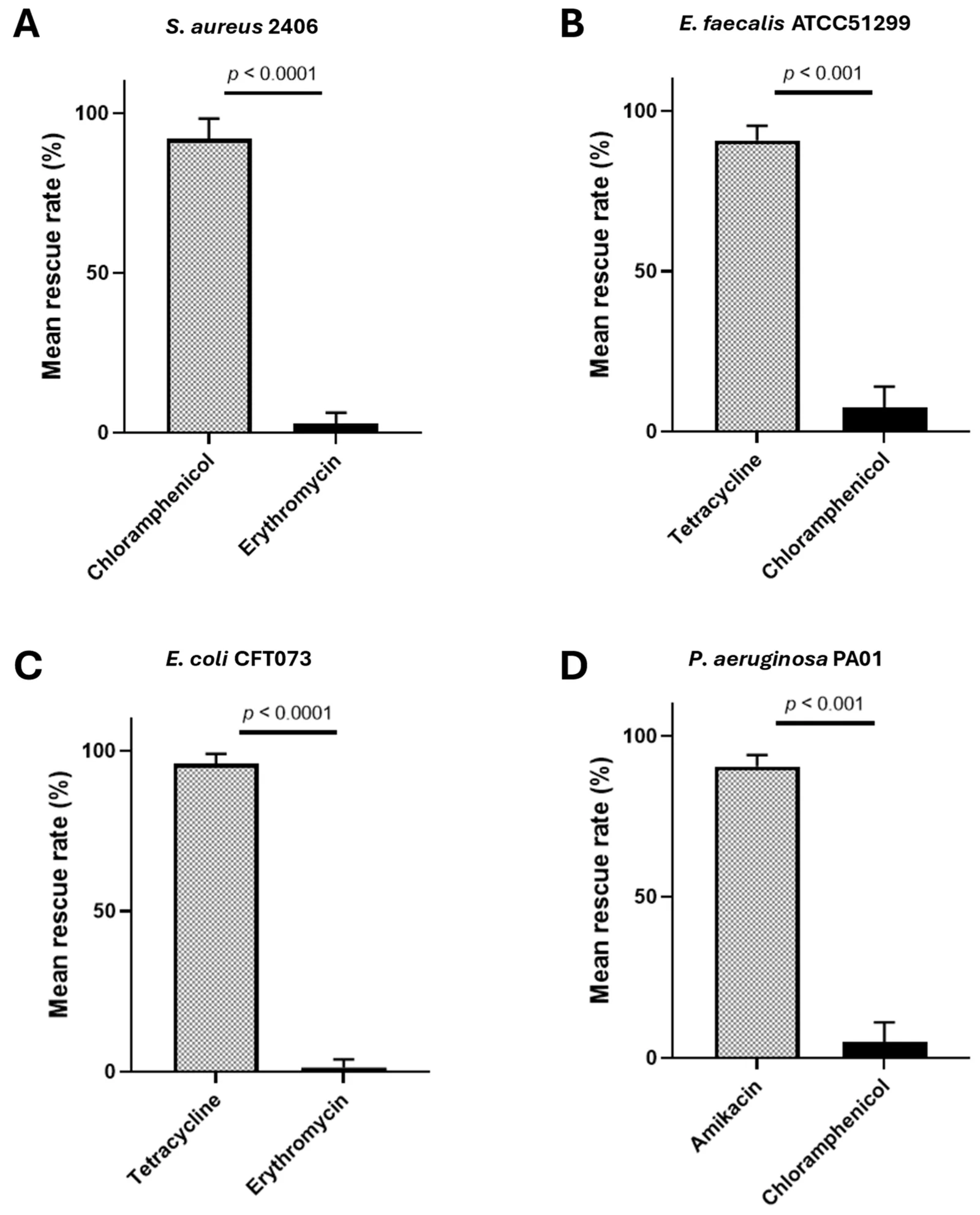
Validation of the *Caenorhabditis elegans* infection–rescue assay using positive- and negative-control antibiotics. Mean rescue rate (MRR) of AU37 mutant *C. elegans* infected with (**A**) *Staphylococcus aureus* 2406, (**B**) *Enterococcus faecalis* ATCC 51299, (**C**) *Escherichia coli* CFT073, and (**D**) *Pseudomonas aeruginosa* PAO1 following treatment with positive- or negative-control antibiotics at the optimized infection durations. Patterned bars indicate positive-control antibiotics, and solid black bars indicate negative-control antibiotics. Bars represent mean MRR ± SD from four independent biological replicates. Reported *p* values compare positive- and negative-control antibiotic MRR values within each pathogen.

**Figure 3:**
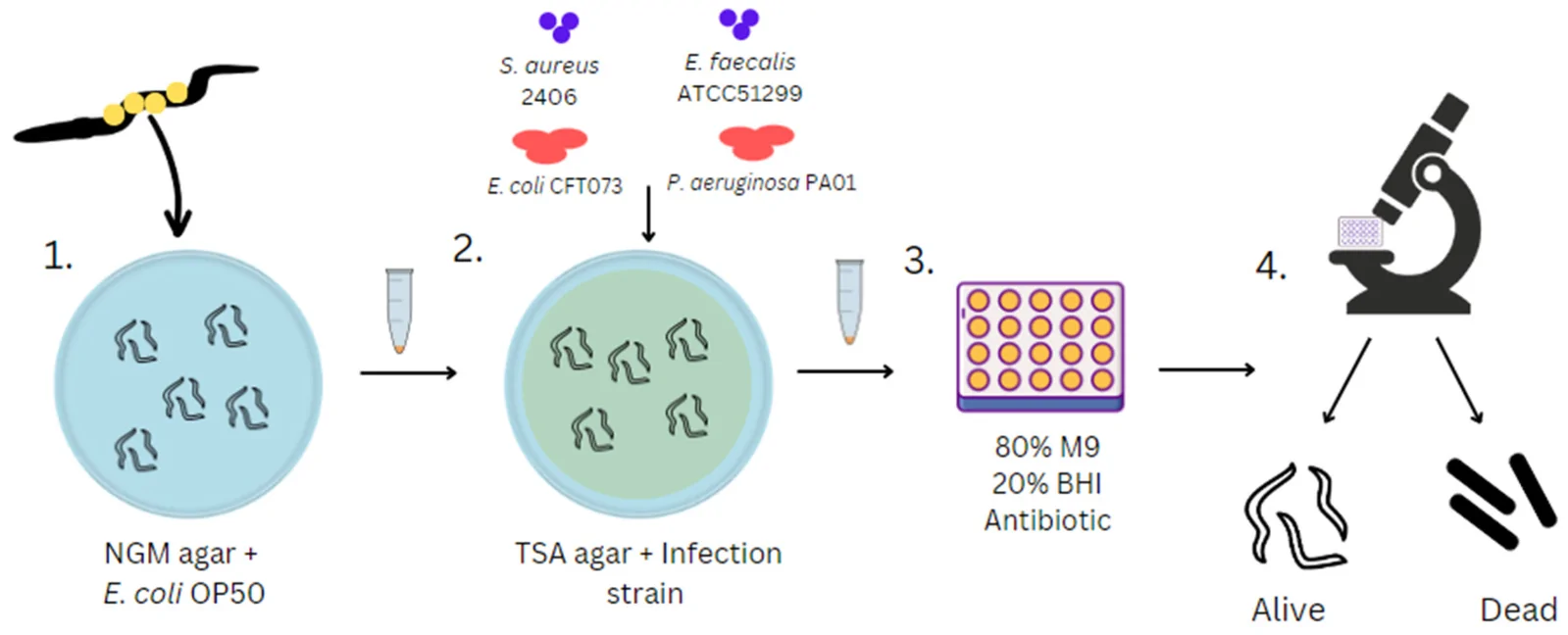
Schematic overview of the *C. elegans* antibiotic screening workflow. (1) Gravid adult hermaphrodites were synchronized to obtain eggs, which were grown to the L4 larval stage on nematode growth medium (NGM) seeded with *Escherichia coli* OP50. (2) L4 worms were transferred to tryptic soy agar (TSA) plates containing the pathogen of interest and nalidixic acid (10 µg/mL) to suppress OP50 growth. (3) Infected worms were transferred to 24-well plates containing liquid media (80% M9 buffer, 20% BHI broth) supplemented with the appropriate antibiotic and nalidixic acid. (4) Worm survival was assessed after 24 h using a stereomicroscope. Worms were scored as dead if they displayed a rigid straight morphology and as alive if sinusoidal movement was observed.

**Table 1:** Assay-specific growth inhibition and working antibiotic concentrations used for infection–rescue optimization.

Pathogen	Antibiotic	Growth-Inhibitory Concentration (µg/mL)	Working Concentration Used in Infection–Rescue Assay (µg/mL)
*S. aureus* 2406	Vancomycin	5	10
*E. faecalis* ATCC 51299	Gentamicin	25	50
*E. coli* CFT073	Gentamicin	25	50
*P. aeruginosa* PAO1	Gentamicin	6.25	12.5

Note: growth-inhibitory concentrations were determined under assay-specific broth microdilution conditions and were used to select working antibiotic concentrations for the *C. elegans* infection–rescue assay. These values were not intended to define formal MIC or clinical susceptibility breakpoints.

**Table 2:** Antibiotic control pairs and concentrations used for validation of the *C. elegans* infection–rescue assay.

Pathogen	Positive-Control Antibiotic	Susceptibility Profile	Concentration Used (µg/mL)	Negative-Control Antibiotic	Susceptibility Profile	Concentration Used (µg/mL)	Optimized Infection Duration (h)
*S. aureus* 2406	Chloramphenicol	Susceptible	6.25	Erythromycin	Resistant	6.25	48
*E. faecalis* ATCC51299	Tetracycline	Susceptible	12.5	Chloramphenicol	Resistant	12.5	72
*E. coli* CFT073	Tetracycline	Susceptible	12.5	Erythromycin	Resistant	12.5	4
*P. aeruginosa* PAO1	Amikacin	Susceptible	12.5	Chloramphenicol	Resistant	12.5	72

Note: Positive- and negative-control antibiotics were selected based on contrasting Kirby–Bauer disk diffusion susceptibility profiles for each bacterial strain. Concentrations indicate the working antibiotic concentrations used in the *C. elegans* infection–rescue validation assay. These antibiotic–pathogen pairs were selected to evaluate assay performance and should not be interpreted as clinically recommended treatment combinations.

**Table 3:** Comparison of antimicrobial screening platforms.

Screening Platform	Advantages	Limitations	Use in Antimicrobial Screening
Conventional in vitro growth inhibition assays	Rapid, inexpensive, standardized, high-throughput	Do not capture host toxicity, bioavailability, or infection context	First-line screening for antibacterial activity
Cell culture infection assays	Include host cells and intracellular effects	Limited whole-organism physiology	Useful for host-cell-associated antimicrobial effects
Mammalian infection models	High physiological relevance	Expensive, low-throughput, ethically constrained	Later-stage validation of antimicrobial efficacy
Existing *C. elegans* virulence models	Whole-animal model for pathogen-mediated killing	Often focus on virulence rather than antibiotic rescue	Useful for studying infection dynamics
Current *C. elegans* infection–rescue assay	Rapid, scalable, live-host rescue readout	Requires pathogen-specific optimization; limited mammalian pharmacokinetics	Intermediate platform for early antimicrobial screening

## Data Availability

All data generated or analyzed during this study are included in this article and its Supplemental Materials.

## Supplementary Materials

The following supporting information can be downloaded at: https://www.mdpi.com/article/10.3390/antibiotics15100946/s1, Supplementary Table S1: Kirby–Bauer antibiotic susceptibility profiles of selected bacterial strains; Supplementary Table S2: Assay-specific broth microdilution growth-inhibition results for conventional antibiotics used during infection-duration optimization; Supplementary Table S3: Assay-specific broth microdilution growth-inhibition results for positive- and negative-control antibiotics used for assay validation; Supplementary Table S4: Nalidixic acid growth-inhibition testing against bacterial strains used in the *C. elegans* infection–rescue assay.

## Abbreviations

The following abbreviations are used in this manuscript:

AMR	antimicrobial resistance
MDR	multidrug-resistant
MRSA	methicillin-resistant *Staphylococcus aureus*
VRE	vancomycin-resistant enterococci
MIC	minimum inhibitory concentration
MRR	mean rescue rate
CLSI	Clinical and Laboratory Standards Institute
NGM	nematode growth medium
TSA	tryptic soy agar
BHI	brain heart infusion
OP50	*Escherichia coli* OP50 food strain
AU37	*Caenorhabditis elegans glp-4*; *sek-1* mutant strain
