# Supplementary material for: Caenorhabditis elegans as an In Vivo Infection–Rescue Model for Early-Stage Antimicrobial Screening Against Gram-Positive and Gram-Negative Bacteria
Source: Antibiotics (Basel). 2026 Sep 23;15(10):946. doi: 10.3390/antibiotics15100946 (PMC13700891; doi:10.3390/antibiotics15100946)
Supplement: Supplementary file 1 [file antibiotics-15-00946-s001.zip › antibiotics-4570092-supplementary.pdf]

Supplementary Table S1. Kirby–Bauer antibiotic susceptibility profiles of selected bacterial strains.

	Kirby-Bauer susceptibility assay			
Antibiotic	*S. aureus* 2406	*E. faecalis* ATCC51299	*E. coli* CFT073	*P. aeruginosa* PAO1
Nitrofurantoin	S	S	S	R
Ciprofloxacin	S	S	S	R
Chloramphenicol	S	R	S	**R**
Penicillin	R	S	R	R
Doxycycline	S	S	S	R
Erythromycin	R	S	**R**	R
Tetracycline	S	S	**S**	R
Amikacin	S	R	S	S
Gentamicin	S	S	S	S
Levofloxacin	I	S	S	S
Linezolid	S	S	R	R

Note: S, susceptible; I, intermediate; R, resistant. Susceptibility classifications were interpreted using the CLSI-based zone diameter interpretive criteria provided with the antibiotic disk reference chart. Bold red entries indicate the positive- and negative-control antibiotics selected for *C. elegans* infection–rescue assay validation.

Supplementary Table S2. Assay-specific broth microdilution growth-inhibition results for conventional antibiotics used during infection-duration optimization.

Vancomycin		Gentamicin			
S. aureus (2406)	Concentration (µg/mL)	*E. faecalis* (ATCC51299)	*E. coli* (CFT073)	*P. aeruginosa* (PA01)	Concentration (µg/mL)
+	1.25	+	+	+	3.125
+	2.50	+	+	-	6.25
-	5.0	+	+	**-**	12.50
**-**	10.0	-	-	-	25.0
-	20.0	**-**	**-**	-	50.0

Note: Symbols indicate bacterial growth (+) or inhibition (−) at the indicated antibiotic concentrations. Bold red entries indicate the working antibiotic concentrations selected for subsequent *C. elegans* infection–rescue experiments. These values were used for assay optimization and do not define clinical susceptibility breakpoints.

Supplementary Table S3. Assay-specific broth microdilution growth-inhibition results for positive- and negative-control antibiotics used for assay validation

	***S. aureus* (2406)**		***E. faecalis* (ATCC51299)**		***E. coli* (CFT073)**		***P. aeruginosa* (PA01)**	
	S	R	S	R	S	R	S	R
MIC (ug/ml)	CM	ERY	TET	CM	TET	ERY	AMIK	CM
3.125	-	+	-	+	-	+	-	+
6.25	**-**	**+**	-	+	-	+	-	+
12.5	-	-	**-**	**+**	**-**	**+**	**-**	**+**
25.0	-	-	-	+	-	+	-	+
50.0	-	-	-	+	-	+	-	+

Note: CM, chloramphenicol; ERY, erythromycin; TET, tetracycline; AMIK, amikacin. S and R indicate antibiotics selected as positive- and negative-control treatments, respectively, based on disk/tablet diffusion susceptibility profiles. Symbols indicate bacterial growth (+) or inhibition (−) at the indicated antibiotic concentrations. Bold red entries indicate the working antibiotic concentrations selected for *C. elegans* infection–rescue assay validation. These values were used for assay validation and do not define clinical susceptibility breakpoints.

Supplementary Table S4. Nalidixic acid growth-inhibition testing against bacterial strains used in the *C. elegans* infection–rescue assay.

**Nalidixic acid** (µg/ml)	*S. aureus* 2406	*E. faecalis* ATCC51299	*E. coli* CFT073	*P. aeruginosa* PA01
5	+	+	-	+
10	+	+	-	+
20	+	+	-	+
40	+	+	-	+

Note: Symbols indicate bacterial growth (+) or inhibition (−) at the indicated nalidixic acid concentrations. Nalidixic acid inhibited *E. coli* CFT073 and was therefore omitted from *E. coli* infection–rescue experiments
